# Whole exome sequencing identified a novel compound heterozygous mutation of *nephrocystin 4* in a child with nephronophthisis—a rare case report

**DOI:** 10.3389/fped.2026.1864993

**Published:** 2026-07-07

**Authors:** Wang Li, Gao-Hui Cao, Nan-Nan Li, Jie-Yi Long, You-Qing Tang, Ji-Shi Liu, Liang-Liang Fan

**Affiliations:** 1 Department of Emergency Medicine, Guangdong Second Provincial General Hospital, Third Clinical College of Jinan University, Guangzhou, China; 2 Department of Cell Biology, School of Life Sciences, Central South University, Changsha, China; 3 Department of Nephrology, The Third Xiangya Hospital of Central South University, Changsha, China

**Keywords:** compound heterozygous mutation, nephronophthisis, NPHP, NPHP4 gene, proteinuria

## Abstract

Nephronophthisis (NPHP), an autosomal recessive kidney disorder characterized by chronic tubulointerstitial nephritis, is the most prevalent monogenic cause of end-stage renal disease (ESRD). Previous studies have identified variants in more than 20 genes, designated *NPHP1* to *NPHP20*, and in additional loci as the genetic causes of NPHP. Here, a Chinese patient who presented with NPHP and proteinuria was enrolled. By employing whole exome sequencing and Sanger sequencing, we identified a rare compound heterozygous mutation in the *NPHP4* gene (c.2611C > T/p.R871X and c.2768G > A/p.R923H) in the patient. Sanger sequencing further confirmed that the p.R871X variant was inherited from the father and that the p.R923H variant originated from the mother. Both variants were predicted to be deleterious by bioinformatic software. In accordance with the American College of Medical Genetics and Genomics guideline, the p.R871X variant was classified as pathogenic, and the p.R923H variant was classified as likely pathogenic. So far, neither of these mutations has been reported in patients with NPHP. Our findings contribute to the precise diagnosis of the patient and further underscore the utility of genetic testing in the accurate diagnosis of NPHP.

## Introduction

Nephronophthisis (NPHP), a chronic tubulointerstitial nephritis, is characterized by progressive fibrosis and cystic changes predominantly affecting the renal medulla, ultimately leading to end-stage renal disease (ESRD) (1). The disorder, first described by Smith and Graham Fanconi (2), is currently classified into three forms on the basis of the age of onset of ESRD: infantile, juvenile, and adolescent (3). Juvenile NPHP is the most prevalent form in the pediatric ESRD population, and the onset of ESRD in these patients occurs at a median age of 12 years (4). The hallmark clinical features of NPHP include polyuria, polydipsia, growth retardation, and anemia (5). Additional manifestations, such as fatigue, weakness, nausea, bone pain, and hypertension, may also be present in affected individuals (6). Currently, the incidence rate of NPHP is estimated to range from 1 in 50,000 to 1 in 1,000,000 individuals (7).

Nephronophthisis (NPHP) is a rare autosomal recessive disorder classified within the group of ciliopathies, indicating that defects in the structure and function of primary cilia are central to its pathogenesis (8, 9). Primary cilia are slender, antenna-like organelles present on the surface of most mammalian cells, including renal tubular epithelial cells (1). These structures serve as critical sensory hubs that regulate key signaling pathways essential for embryonic development and tissue homeostasis (1). Dysfunction of primary cilia disrupts vital signaling cascades, such as the Wnt and Hedgehog pathways, resulting in aberrant cellular differentiation, impaired tissue architecture, and progressive organ damage (8, 9). To date, mutations in more than 20 genes, designated *NPHP1* through *NPHP20* and in additional loci, have been identified as causative genetic variants in NPHP, all of which encode proteins involved in ciliary biology or function (4). Among these, *nephrocystin 1* (*NPHP1*) mutations represent the most prevalent genetic cause, accounting for approximately 20%–40% of juvenile-onset cases (10, 11).

The human *NPHP4* gene (NM_015102), which encodes the nephrocystin-4 protein, is located on chromosome 1p36.31 and comprises 30 exons spanning approximately 129 kilobases (kb). Previous studies have demonstrated that NPHP4 is essential for the formation of functional cilia, thereby influencing renal tubular development (11). Additionally, it plays a critical role in organizing the subapical actin cytoskeleton in multiciliated epithelial cells (12). In 2002, Mollet et al. and Otto et al. independently reported that biallelic mutations in *NPHP4* are associated with NPHP (10, 11). To date, more than 140 pathogenic variants in *NPHP4* have been identified in patients with NPHP and Senior-Løken syndrome, an autosomal recessive disorder characterized by the combination of NPHP and retinal degeneration (13).

## Case presentation

**Figure 1:**
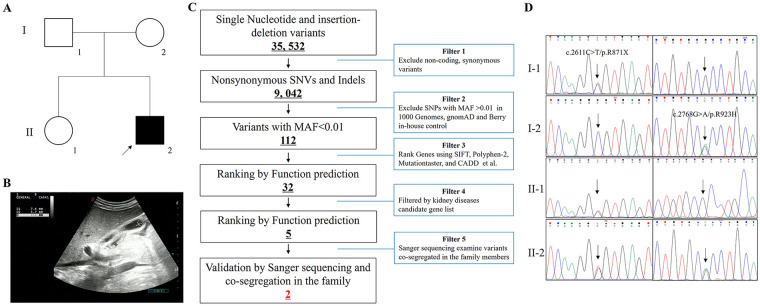
The clinical data and genetic sequencing of the patient. **(A)** Pedigree of the family. Black circles/squares are affected; white circles/squares are unaffected. Arrow indicates the proband. **(B)** The B ultrasonic data of the patient. **(C)** Schematic representation of the filter strategies employed in our study. **(D)** Sanger DNA sequencing chromatogram demonstrates a compound heterozygous mutation (c.2611C > T/p.R871X and c.2768G > A/p.R923H) in the patient.

An 11-year-old male was admitted to the hospital because of generalized edema that had persisted for more than 10 days (Figure 1A). Physical examination before admission revealed facial and conjunctival edema, as well as edema involving the extremities and scrotum. Vital signs, including body temperature and blood pressure, along with electrocardiogram findings and thoraco-abdominal percussion results, were within normal limits. Following the patient's admission to the hospital, a series of relevant diagnostic evaluations were conducted. Urinalysis revealed a urine specific gravity of 1.004 (reference range: 1.015–1.025), positive proteinuria (+++, 3.0 g/L), occult blood +1, a white blood cell count of 36.5 × 10^9^/L, a red blood cell count of 1.164 × 10^12^/L, and a 24-hour urinary protein excretion of 4,704 mg (normal <150 mg). Renal function tests revealed elevated levels of urea (13.56 mmol/L; reference range: 2.8–7.1 mmol/L), creatinine (151 μmol/L; reference range: 53–106 μmol/L), and uric acid (540 μmol/L; reference range: 208–428 μmol/L). The procalcitonin concentration was elevated to 0.64 ng/mL (reference range: <0.05 ng/mL). The level of C-reactive protein is 21.4 mg/L (reference range: <8.0 mg/L) and the measurement of erythrocyte sedimentation rate is 55 mm/h (reference range: <20 mm/h). The parathyroid hormone concentration was elevated to 170.2 pg/mL (reference range: 15–65 pg/mL), and the creatinine clearance rate was decreased to 42.2 mL/min (reference range: 80–120 mL/min). Additional laboratory assessments, including a complete blood count, liver function tests, and a lipid profile, were within normal limits. Abdominal color Doppler ultrasonography revealed the following renal measurements: left kidney, 98 × 48 mm; right kidney, 91 × 43 mm. Both kidneys exhibited regular morphology and well-defined contours. Parenchymal echogenicity was mildly increased bilaterally, with no evidence of focal masses or calculi (Figure 1B). Medical investigations revealed that the patient began to experience fatigue, loss of appetite, and lower back pain one year prior, along with symptoms such as nocturnal enuresis and frequent urination at night. The family history indicated that the patient's parents had no significant medical conditions, and the patient's sister (II-1) had no history of related kidney diseases (Figure 1A). The main clinical manifestations in this patient were edema, enuresis, polydipsia, polyuria, anemia, and renal dysfunction, which are consistent with the typical characteristics of NPHP. Additionally, this patient exhibited an atypical feature of increased urine protein. The patient's condition improved following treatment with prednisone, tacrolimus, compound *α*-ketoacid, dipyridamole, cordyceps capsules and shenyankangfu tablets. The patient declined to undergo renal biopsy. The physician recommended a follow-up examination one month after initiation of pharmacotherapy. However, the patient declined subsequent care at our institution.

Genomic DNA was extracted from peripheral blood lymphocytes of the patient and his parents using the DNeasy Blood & Tissue Kit (Qiagen, Valencia, CA) according to the manufacturer's instructions. SNP-array examination did not uncover any structural or numerical variations in chromosomes. Consequently, whole exome sequencing was employed to investigate the underlying variants of the proband as we previous described (14). Exome capture was carried out using the Agilent SureSelect Human All Exon V6 kit, followed by high-throughput sequencing on an Illumina HiSeq 4,000 platform. Targeted capture, massive parallel sequencing, read alignment, variant calling, initial data filtering, and annotation were conducted by the Berry Genomics Institute (Beijing, China). Candidate variants identified through filtering were validated by Sanger sequencing, and co-segregation analysis was performed within the family. Primer pairs were designed using Primer 5 software (primer sequences available upon request). PCR products were analyzed using the ABI 3,100 Genetic Analyzer (Applied Biosystems, Foster City, CA). Bioinformatics analyses were conducted as described in our previous study (14).

**Figure 2:**
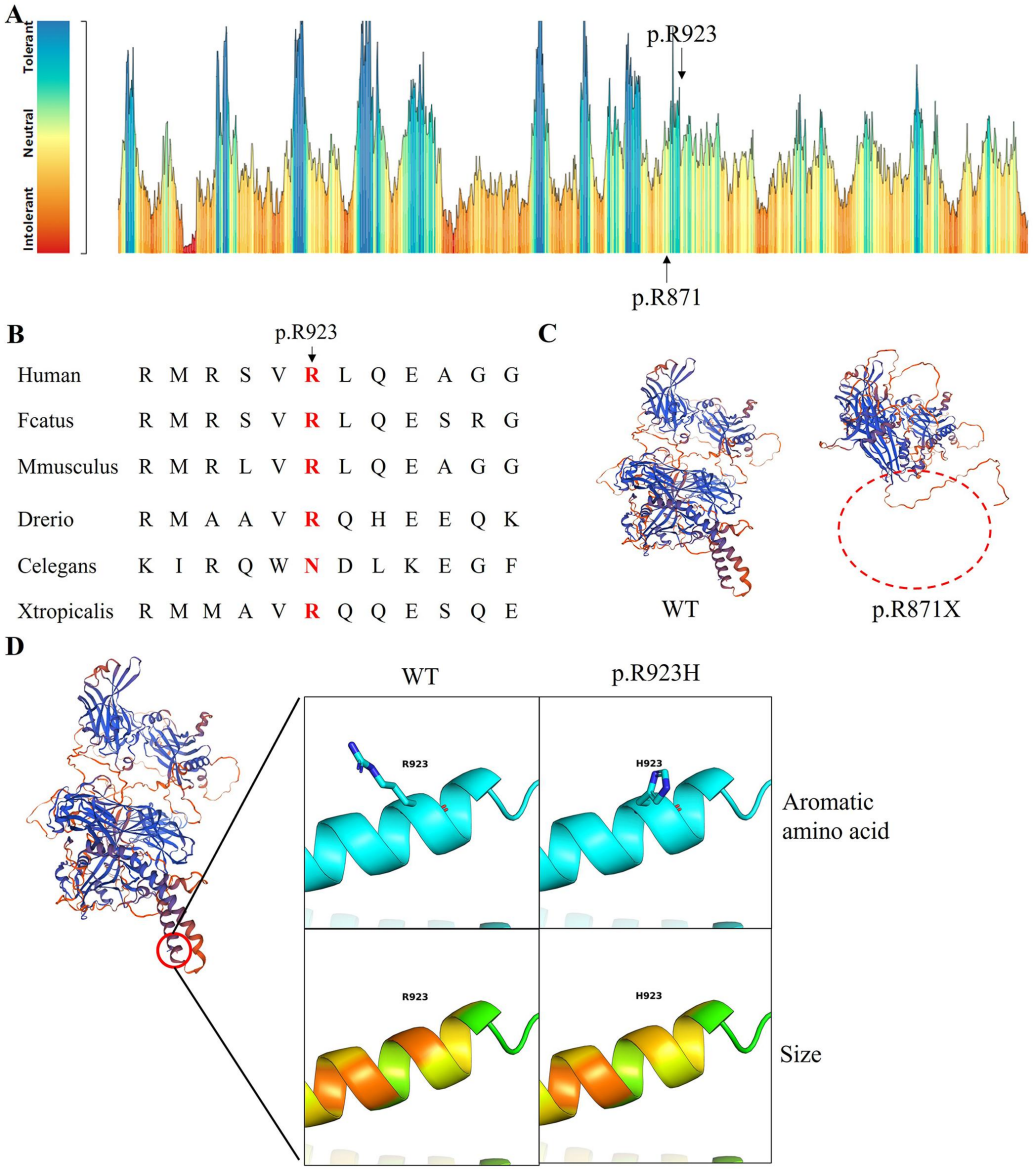
The genetic analysis of the compound heterozygous mutation. **(A)** Intolerance analysis of the NPHP4 amino acid sequence revealed that both variants position reside within genomic regions highly intolerant to missense variation. **(B)** A conservation analysis of the NPHP4 protein's amino acid sequence revealed that the p.R923H mutation site has high conservation. **(C)** the p.R871X mutation was predicted to disrupt the NPHP4 protein architecture by Swiss-model program. **(D)** the p.R923H substitution was predicted to disrupt the *α*-helical conformation and alters size of the NPHP4 protein.

Whole exome sequencing produced 9.20 Gb of data with coverage of 99.5% of the target regions; 98.1% of these regions were covered more than 10 times. Following alignment and single nucleotide variant calling, a total of 71,461 variants were identified in the proband. Following the data filtering strategy of whole exome sequencing combined with kidney diseases candidate gene list filtering (Figure 1C, Supplementary Table S1), two rare *NPHP4* variants (c.2611C > T/p.R871X and c.2768G > A/p.R923H), previously associated with NPHP, were identified in the proband (Supplementary Table S1). Sanger sequencing confirmed that the nonsense variant (c.2611C > T/p.R871X) was present in the proband, his father, and his sister, while the missense variant (c.2768G > A/p.R923H) was detected in both the proband and his mother (Figure 1D). Neither variant was observed in our local cohort of 200 controls or in public databases, including 1,000 Genomes (1000G), dbSNP147, and ESP6500. Both variants exhibited extremely low minor allele frequencies (MAFs) in gnomAD (p.R871X: 0.00000479; p.R923H: 0.00000693). Both mutations are located in functionally intolerant regions of the NPHP4 protein (Figure 2A), with the missense mutation affecting an evolutionarily highly conserved residue (Figure 2B). *In silico* analyses predicted that both variants were deleterious. Protein structure modeling revealed that the p.R871X variant led to premature truncation and complete disruption of the NPHP4 protein architecture (Figure 2C), whereas the p.R923H substitution altered the size of the protein (Figure 2D). In accordance with the American College of Medical Genetics and Genomics (ACMG) guidelines, the p.R871X variant is classified as pathogenic, fulfilling the following criteria: PVS1 + PM2 + PP1 + PP3. The p.R923H variant is classified as likely pathogenic based on the following criteria: PM2 + PM3 + PP1 + PP3.

## Discussion

NPHP is a recognized cause of unexplained renal failure in children and adolescents, invariably progressing to end-stage renal disease (4). The disease is characterized by an insidious onset and nonspecific clinical manifestations, making early diagnosis challenging (15). Even renal biopsy often fails to establish a definitive diagnosis. Therefore, NPHP should be considered in pediatric and adolescent patients who present with unexplained kidney injury, particularly when it is accompanied by abnormalities in the retina, central nervous system, or skeletal development (15). At present, high-throughput sequencing has emerged as an effective diagnostic tool for kidney diseases. For example, Bakhtawar Farooq et al. successfully identified the genetic cause of a 4-year-old girl with chronic kidney disease through whole exome sequencing (16). Currently, genetic testing is recommended as the first-line approach for confirming NPHP in developed countries, including those in Europe, the United States, and Canada (17, 18). In this study, we investigated an adolescent patient who presented with NPHP and proteinuria. Through whole-exome sequencing followed by Sanger validation, we identified a novel compound heterozygous mutation in the *NPHP4* gene (c.2611C > T/p.R871X and c.2768G > A/p.R923H). These findings enabled a precise molecular diagnosis of Nephronophthisis type 4, thereby facilitating accurate clinical management and informing subsequent therapeutic strategies.

The nephrocystin-4 protein, consisting of 1,426 amino acids, comprises three distinct domains: an N-terminal predicted coiled-coil domain, a Src homology 3 (SH3) domain, and a highly conserved C-terminal nephrocystin homology domain (NHD) (10). The N-terminal domain mediates protein‒protein interactions, whereas the SH3 domain is involved in signaling pathways that regulate cell adhesion and cytoskeletal organization (10, 19). The NHD (residues 823–1,426) plays a critical role in the localization of proteins to the ciliary basal body and is essential for the proper trafficking and localization of specific proteins within cilia (10, 19). Notably, the c.2611C > T/p.R871X and c.2768G > A/p.R923H variants in *NPHP4* are both located within the NHD region. These mutations may disrupt the structural integrity and functional activity of cilia, particularly impairing protein transport in renal tubular epithelial cell cilia, ultimately leading to ciliary dysfunction and NPHP. This study identifies the genetic etiology in the affected patient and expands the known mutational spectrum of the *NPHP4* gene.

As a known causative gene for ciliopathies, NPHP4 plays a critical role in regulating ciliary function (10, 12). Specifically, NPHP4 recruits KIF13B to the ciliary base, thereby facilitating Sonic Hedgehog signaling (20). A *C. elegans* study revealed that *nphp4* mutations can disrupt the interaction between nphp4 and the MKS (Meckel–Gruber syndrome) complex, further altering ciliary function (21). Studies in *Xenopus laevis* epidermal multiciliated cells have demonstrated that *nphp4* knockdown impairs ciliogenesis and disrupts directional fluid flow (22). Zebrafish models further confirm that nphp4 is essential for the development of motile cilia in Kupffer's vesicle, which generates the asymmetric fluid flow required for proper left–right (L-R) axis patterning (22). In *nphp4*-deficient cells, the ciliary localization of specific membrane proteins is significantly reduced, and cellular housekeeping proteins larger than 50 kDa are no longer effectively excluded from the cilium (23). These findings indicate that NPHP4 functions at the ciliary transition zone as a key component of a selective barrier that regulates both the membrane and soluble protein composition of cilia. In the present study, the patient with *NPHP4* mutations exhibited NPHP, which was likely due to impaired barrier function at the ciliary transition zone (24).

Juvenile NPHP is typically characterized by polydipsia, polyuria, enuresis, growth retardation, chronic anemia, and progressive chronic kidney disease, often in the absence of hypertension (25). Renal ultrasonography typically reveals increased cortical echogenicity, indistinct corticomedullary differentiation, and cysts localized to the renal medulla or at the corticomedullary junction. In the present case, the proband exhibited anemia, polydipsia, polyuria, peripheral edema, and impaired renal function, which were consistent with classic NPHP morphology. Notably, significant proteinuria represented an atypical feature. Previous studies in Japanese patients with nephronophthisis have also reported proteinuria in carriers of pathogenic variants in *NPHP1*, *NPHP3* and *NPHP4* (26). In 2023, Adele Mitrotti et al. also reported that in a consanguineous family, multiple siblings harboring biallelic pathogenic variants in the *NPHP4* gene presented with persistent proteinuria and progressive chronic kidney disease. Renal biopsy performed in one affected individual confirmed a diagnosis of familial glomerulonephritis (27). A key limitation of this study was the unavailability of renal biopsy specimens from the patient, which precluded histopathological correlation and definitive subclassification of the glomerular lesion. The observed proteinuria in individuals with NPHP4-associated nephropathy may reflect one or more of the following mechanisms: (1) compensatory hyperfiltration in residual nephrons secondary to progressive loss of functional renal mass; (2) intrinsic podocyte dysfunction which potentially linked to disrupted cilia-dependent signaling pathways critical for podocyte cytoskeletal integrity and slit diaphragm maintenance (27); or (3) transient alterations in glomerular permeability attributable to concurrent infection or systemic edema (28).

To date, patients harboring biallelic pathogenic variants in *NPHP4* have a median age of progression to ESRD of 16.0 years (29). Approximately 25% progress to ESRD before age 11, whereas another 25% remain dialysis-free beyond age 25 (29). Large-scale cohort studies have indicated that renal transplantation outcomes in NPHP patients, including those with NPHP4-associated disease, are generally superior to those observed in broader pediatric transplant populations (30). However, a Korean cohort study revealed that among six patients with NPHP4-related Senior-Løken syndrome, two developed progressive ESRD despite successful kidney transplantation (31). Given the predictable trajectory of renal decline in NPHP4-associated disease and the favorable transplantation prognosis documented in the literature, preemptive kidney transplantation (32), defined as transplantation prior to the initiation of maintenance dialysis, is strongly recommended for this patient.

Patients harboring *NPHP4* mutations exhibit considerable phenotypic variability (4). The majority of affected individuals present with NPHP, either in isolation or in association with retinal degeneration (15). Additional clinical features observed in individuals with *NPHP4* mutations include infertility, intellectual disability, and congenital heart malformations (10). However, other phenotypes, including heart malformations and Senior-Loken syndrome, were detected only in a few patients, and the correlation between NPHP4 domain-specific mutations and clinical phenotypic heterogeneity has not been established. Notably, even among siblings within the same family who share identical mutations, there can be marked differences in the age of onset and rates of renal disease progression (4, 10). This phenotypic diversity highlights the genetic heterogeneity characteristic of ciliopathies (15). Furthermore, environmental factors are believed to contribute significantly to the pathogenesis and clinical expression of these disorders. In this report, we describe a patient with biallelic *NPHP4* mutations who exhibited classic features of NPHP, further supporting the critical role of the NPHP4 protein in maintaining normal renal function.

In summary, we identified a rare compound heterozygous mutation in the *NPHP4* gene (c.2611C > T/p.R871X and c.2768G > A/p.R923H) in a patient with NPHP through whole exome sequencing followed by Sanger sequencing validation. This finding not only confirmed the clinical diagnosis and expanded the existing mutational spectrum of the *NPHP4* gene, but also further supported the utility of genetic testing as an effective diagnostic tool for NPHP.

## Data Availability

The original contributions presented in the study are included in the article/Supplementary Material, further inquiries can be directed to the corresponding authors.
