# Supplementary material for: Whole exome sequencing identified a novel compound heterozygous mutation of nephrocystin 4 in a child with nephronophthisis—a rare case report
Source: Front Pediatr. 2026 Jul 7;14:1864993. doi: 10.3389/fped.2026.1864993 (PMC13385333; doi:10.3389/fped.2026.1864993)
Supplement: Supplementary file 1 [file Table1.docx]

Table S1. The list of candidate genes in CKD

| BMP4 | WNT4 | COQ2 | PDSS2 | CD46 | SDCCAG8 | CA2 | SLC4A1 | TMEM231 | MKKS | SLC7A7 | WNK4 |
| --- | --- | --- | --- | --- | --- | --- | --- | --- | --- | --- | --- |
| CHD1L | ACE | COQ6 | PLCE1 | C3 | NPHP10 | CASR | SLC7A9 | CSPP1 | BBS7 | SLC4A4 | KLHL3 |
| DSTYK | AGT | CRB2 | PTPRO | COL4A5 | TMEM67 | CLCN5 | SLC9A3R1 | PDE6D | TTC8 | PHEX | CUL3 |
| EYA1 | AGTR1 | CUBN | SCARB2 | COL4A6 | NPHP11 | CLCNKB | VDR | MKS1 | PTHB1 | FGF23 | KCNJ10 |
| GATA3 | CHRM3 | DGKE | SMARCAL1 | NPHP1 | TTC21B | CLDN16 | XDH | TCTN2 | BBS10 | ENPP1 | AVPR2 |
| HNF1B | FGF20 | EMP2 | WDR73 | JBTS4 | NPHP12 | CLDN19 | FGFR1 | B9D1 | TRIM32 | DMP1 | AQP2 |
| MUC1 | FRAS1 | FAT1 | XPO5 | NPHP2 | WDR19 | CYP24A1 | DIS3L2 | B9D2 | BBS12 | CTNS |  |
| PAX2 | FREM1 | ITGA3 | ACTN4 | INVS | NPHP13 | FAM20A | GPC3 | IFT80 | WDPCP | SLC2A2 |  |
| RET | FREM2 | ITGB4 | ANLN | NPHP3 | ZNF423 | GRHPR | OFD1 | DYNC2H1 | LZTFL1 | CLNCKA |  |
| ROBO2 | GRIP1 | KANK1 | ARHGAP24 | NPHP4 | NPHP14 | HNF4A | PKD1 | NEK1 | ALMS1 | BSND |  |
| SALL1 | HPSE2 | KANK2 | INF2 | IQCB1 | CEP164 | HOGA1 | PKD2 | WDR35 | IFT122 | SLC12A3 |  |
| SIX1 | ITGA8 | KANK4 | LMX1B | NPHP5 | NPHP15 | HPRT1 | PKHD1 | WDR60 | IFT43 | TRPM6 |  |
| SIX2 | LRIG2 | LAMB2 | MYH9 | CEP290 | ANKS6 | KCNJ1 | INPP5E | IFT140 | PAX6 | FXYD2 |  |
| SIX5 | REN | MTTL1 | TRPC6 | NPHP6 | NPHP16 | OCRL | TMEM216 | IFT172 | GLA | EGF |  |
| SOX17 | TRAP1 | MYO1E | WT1 | GLIS2 | ADCY10 | SLC12A1 | AHI1 | WDR34 | FGA | CNNM2 |  |
| SRGAP1 | KAL1 | NPHS1 | CFI | NPHP7 | SAC | SLC22A12 | CC2D2A | BBS1 | LYZ | KCNA1 |  |
| TBX18 | ADCK4 | NPHS2 | CFHR5 | RPGRIP1L | AGXT | SLC2A9 | TMEM237 | BBS2 | APOA1 | SCNN1G |  |
| TNXB | ARHGDIA | NUP93 | FN1 | NPHP8 | APRT | SLC34A1 | CEP41 | ARL6 | B2M | SCNN1B |  |
| UMOD | CD2AP | NUP205 | COL4A3 | NEK8 | ATP6V0A4 | SLC34A3 | TMEM138 | BBS4 | SLC5A2 | SCNN1A |  |
| UPK3A | CFH | NUP107 | COL4A4 | NPHP9 | ATP6V1B1 | SLC3A1 | TCTN3 | BBS5 | SLC1A1 | WNK1 |  |
